# The Protein-DNA Interface database

**DOI:** 10.1186/1471-2105-11-262

**Published:** 2010-05-18

**Authors:** Tomás Norambuena, Francisco Melo

**Affiliations:** 1 Departamento de Genética Molecular y Microbiología, Facultad de Ciencias Biológicas, Pontificia Universidad Católica de Chile, Alameda 340, Santiago, Chile

## Abstract

The Protein-DNA Interface database (PDIdb) is a repository containing relevant structural information of Protein-DNA complexes solved by X-ray crystallography and available at the Protein Data Bank. The database includes a simple functional classification of the protein-DNA complexes that consists of three hierarchical levels: Class, Type and Subtype. This classification has been defined and manually curated by humans based on the information gathered from several sources that include PDB, PubMed, CATH, SCOP and COPS. The current version of the database contains only structures with resolution of 2.5 Å or higher, accounting for a total of 922 entries. The major aim of this database is to contribute to the understanding of the main rules that underlie the molecular recognition process between DNA and proteins. To this end, the database is focused on each specific atomic interface rather than on the separated binding partners. Therefore, each entry in this database consists of a single and independent protein-DNA interface.

We hope that PDIdb will be useful to many researchers working in fields such as the prediction of transcription factor binding sites in DNA, the study of specificity determinants that mediate enzyme recognition events, engineering and design of new DNA binding proteins with distinct binding specificity and affinity, among others. Finally, due to its friendly and easy-to-use web interface, we hope that PDIdb will also serve educational and teaching purposes.

## Background

The ability of some proteins to bind selectively to DNA constitutes the basis of key cell processes such as RNA transcription, DNA packing, DNA replication, DNA recombination and DNA repair. The understanding of the molecular recognition process that mediates the specific protein-DNA binding selectivity is one of the most interesting challenges in structural biology. To date, there are several hundreds of protein-DNA complexes that have been solved by X-ray crystallography. These experimental structures, deposited at the Protein Data Bank (PDB) [1] and publicly available to the scientific community, constitute a rich source of information to study the different binding modes and the determinants of protein-DNA binding specificity.

To facilitate the investigation about the mechanisms involved in the protein-DNA recognition process, several databases of protein-DNA complexes and associated software have been developed (Additional file 1). Among these resources we find AANT [2], which has statistical information on aminoacid-nucleotide interactions; ProNuC [3], a database that provides a list of atomic contact pairs between proteins and DNA; ProNIT [4], which gathers experimental binding data of protein-nucleic acid complexes that have been described in the literature; NPIDB [5], a database that contains a description of hydrogen bonds and hydrophobic interactions between proteins and nucleic acids; BIPA [6], a database containing several physicochemical features of protein-nucleic acid interfaces and multiple structural alignments of nucleic-acid binding protein families; and 3D-Footprint [7], which provides estimates of binding specificity for all protein-DNA complexes available at the PDB, among other features.

In addition to these resources, that catalogue important information on protein-DNA interactions, the interest has also been put on classification of the complexes. In this respect, we now have a more or less complete vision about the distinct protein architectures and how they bind to DNA. A detailed fold catalogue that describes the complexes in terms of their function and structure is available [8]. This protein view of the protein-DNA interaction has been the overall trend in classifying protein-DNA complexes. On the other hand, Sarai and colleagues have set up a new approach for the classification of protein-DNA complexes, which is based on some DNA features instead of using only protein features [9]. The existence of these somehow separated or independent views of the protein-DNA complexes have prompted us to develop a more complete annotation of the solved protein-DNA complexes by taking into account the interface as a central feature.

The major aim of the new database described here, the Protein DNA Interaction Database (PDIdb), is to contribute to the understanding of the main rules that underlie the molecular recognition process between DNA and proteins. To this end, we have focused on each specific atomic interface rather than on the separated binding partners (*e.g*. protein or DNA molecules alone). Therefore, each entry in this database consists of a single and independent protein-DNA interface, which has been manually inspected and curated by humans, to avoid or minimize any downstream accumulation of errors in subsequent analysis.

We hope this resource will not be only valuable to researchers and software developers working in different areas such as the structure-based prediction of transcription factor binding sites, the engineering of DNA binding proteins and the computer-based prediction of protein-DNA complex three-dimensional structures, but may also serve for educational purposes.

## Construction and Content

### PDB features and interface definition

**Figure 1:**
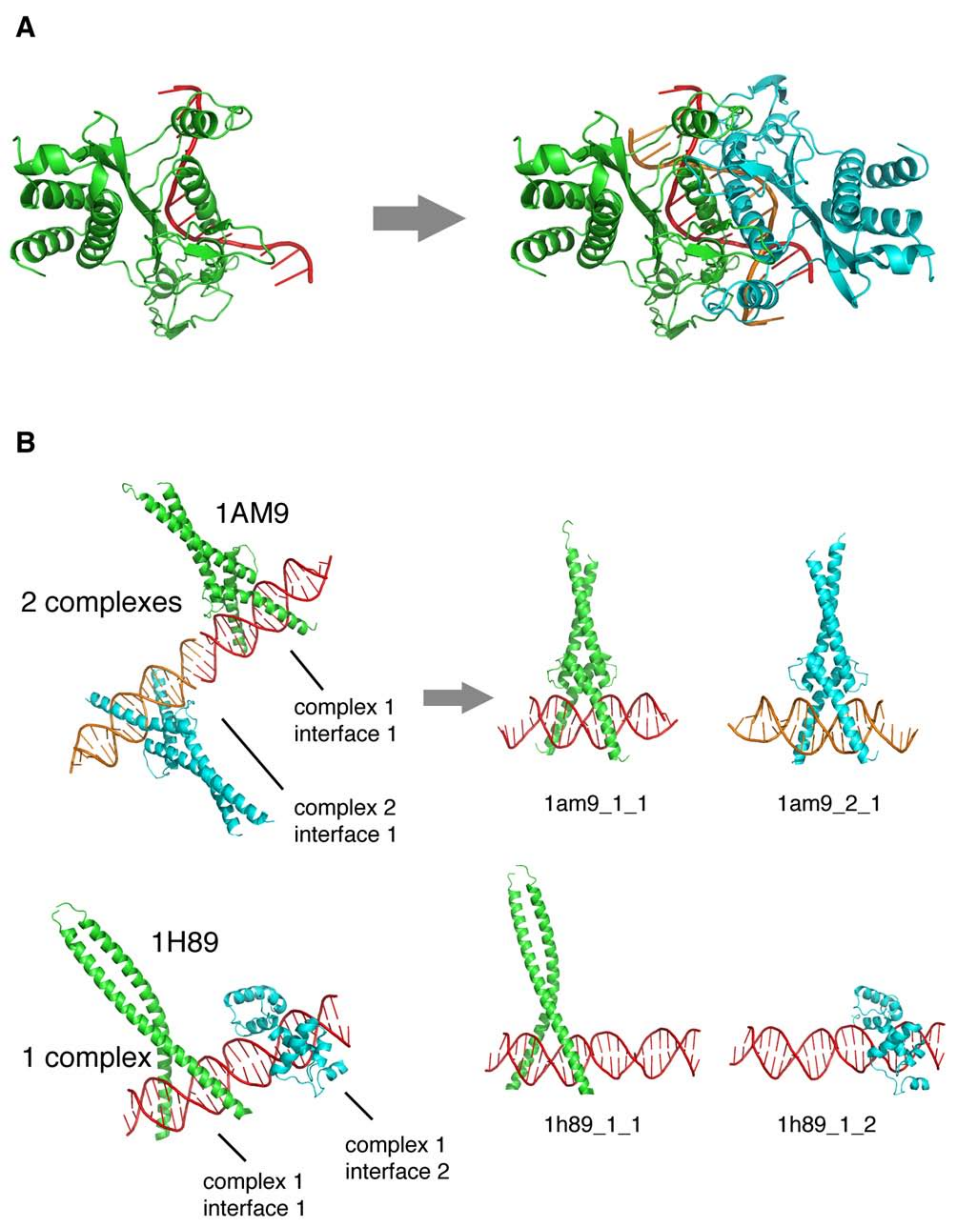
**PDB complexes and interfaces definition**. (A) Example of a structure whose asymmetric unit contained half of the known biological unit (left). For each entry of this type, the complete biological unit was obtained from the specialized ftp site of PDB at ftp://ftp.wwpdb.org. (B) Each entry of the database consists of a single and independent protein-DNA interface, which is isolated from the whole PDB complex. Here, two examples that illustrate this feature are shown. (Top) 1am9, which PDB file contains two separable complexes, each having a single protein-DNA interface; (Bottom) 1h89, which has one complex, but with two independent protein-DNA interfaces. Each interface has assigned a unique ID in the database.

A dataset of protein-DNA complex structures solved by X-ray crystallography was extracted from the PDB in January 12, 2009. The protein-DNA complexes were further selected only if they were solved at a resolution of 2.5 Å or higher. Finally, only those complexes that contained double strand DNA were retained. In addition to this semi-automated filtered search, a manual and visual inspection of all complexes was carried out to determine if their asymmetric and biological units had differences. If this was the case, the corresponding biological units were obtained from a special repository available at the PDB web site in order to have fully-restored structures (Figure 1, panel A).

According to the visual inspection of each complex, the following PDB features were extracted: number of complexes per PDB, which is the number of independent complexes that appear in a PDB entry (sometimes this number matches the number of biological units in the crystal) (Figure 1, panel B); and number of interfaces per complex, which is the number of independent protein-DNA interfaces per complex (Figure 1, panel C).

An interface is defined when one or more protein subunits interacting with DNA can be isolated. For example, the structure with PDB code 1am9 has two complexes, each with one independent interface consisting of a protein dimer interacting with DNA (Figure 1, panel B), while the structure with PDB code 1h89 has one complex with two interfaces (one with a protein monomer interacting with DNA and the other with a protein dimer interacting with DNA) (Figure 1, panel C).

Defined as mentioned above, each interface represents a specific entry of the database and, as such, it is associated with a unique identifier (ID). This ID was constructed by taking into account both the number of complexes per PDB and the number of interfaces per complex. For instance, the structure 1am9 will give rise to two entries with unique identifiers: 1am9_1_1 and 1am9_2_1. The first ID stands for the structure 1am9, complex 1, interface 1; while the second ID stands for the structure 1am9, complex 2, interface 1 (Figure 1, panel B, top). Similarly, the PDB entry 1h89 will be also converted into two independent entries: 1h89_1_1 and 1h89_1_2. In this case, the second ID stands for the structure 1h89, complex 1, interface 2 (Figure 1, panel B, bottom). The enumeration order of the complexes and interfaces is assigned based on the increasing alphabetic chain IDs as recorded in the PDB file.

Other PDB features extracted from the structure inspection were also recorded, which included the resolution, source species information, PubMed ID, the number of biological units, if the asymmetric unit is the same as the biological unit, and if the structure contains oxygen atoms belonging to water molecules.

### Protein features

A simple function/structure-based classification for each entry was defined from the point of view of the protein part of the interface. Following the logic of a previous work [8] and using as a source of information all that available at PubMed, PDB [1], CATH [10], SCOP [11] and COPS [12] databases, we defined three classification categories: **Class**, **Type **and **Subtype **(Table 1). The category **Class **is function-based and contains three subcategories: *Enzyme*, if the main function of the protein is to modify DNA; *Transcription Factor*, if the main function of the protein is to regulate transcription and gene expression; and *Structural/DNA Binding Protein*, if the main function of the protein is to support DNA structure, DNA bending or to aggregate other proteins. The category **Type **is function/structure-based and has 15 subcategories for the *Enzyme ***Class **(Dioxygenase, Endonuclease, Excisionase, Glucosyltransferase, Glycosylase, Helicase, Ligase, Methyltransferase, Nuclease, Photolyase, Polymerase, Recombinase, Topoisomerase, Translocase and Transposase), 7 subcategories for the *Transcription Factor ***Class **(Alpha Helix, Alpha/Beta, Beta Sheet, Helix Turn Helix, Ribbon/Helix/Helix, Zinc Coordinating and Zipper Type), and 8 subcategories for the *Structural/DNA Binding Protein ***Class **(Centromeric Protein, DNA Packaging, Maintenance/Protection, DNA Bending, Repair Protein, Replication, Telomeric Protein and Zalpha). The category **Subtype **involves a more specific classification that takes into account domains, specific reaction of an enzyme, specific DNA binding sites, etc. The detailed description of all three categories can be obtained by quering the database.

**Table 1:** Description of protein features classes and types

Class	Type	Description
Enzyme	Dioxygenase	Enzyme that repairs DNA base lesions by using a direct oxidative dealkylation mechanism [25].
	
	Endonuclease	Restriction enzyme that cleaves DNA at specific sites [26].
	
	Excisionase	Enzyme that controls integrase-mediated DNA rearrangement [27].
	
	Glucosyltransferase	Enzyme that binds DNA in abasic site and flips it. Glucosylation is on a 5-hydroximethylcytosine in duplex DNA using UDP-glucose [28].
	
	Glycosylase	Enzyme involved in base excision repair, a mechanism by which, damaged nucleotides in DNA are removed and replaced. It catalyses the first step in the process [29].
	
	Helicase	Enzyme that unwinds double helices using ATP hydrolysis [30].
	
	Ligase	Enzyme that recognizes nicks and states for strand closure [31].
	
	Methyltransferase	Enzyme responsible for the generation of the genome methylation patterns leading to gene silencing [32].
	
	Nuclease	Enzyme that cleaves DNA, but that are not classified as Endonuclease.
	
	Photolyase	Enzyme that uses light to repair DNA having UV-induced lesions [33].
	
	Polymerase	Enzyme that takes nucleotides from solvent, and catalyses the synthesis of a polynucleotide sequence against a nucleotide template strand using base-pairing interactions [34].
	
	Recombinase	Enzyme that catalyses the reciprocal exchange of DNA strands in the direct site-specific DNA recombination process [35].
	
	Topoisomerase	Enzyme that promotes the relaxation of DNA superhelical lesions by introducing a transient single stranded break in duplex DNA [36].
	
	Translocase	Enzyme that segregates dimeric circular chromosomes, formed by recombination of monomer sisters [37].
	
	Transposase	Enzyme that mediates transposition, a process whereby defined DNA segments move freely about the genome [38].

Structural/DNA Binding	Centromeric Protein	Protein that is part of a chromosome centromere.
	
	DNA Packaging	Protein that is part of the chromosome and packages the DNA.
	
	Maintenance/Protection	Protein involved in the protection and maintenance of the genome.
	
	DNA Bending	Protein that bends DNA with a highly component of indirect readout.
	
	Repair Protein	Protein that recognizes damaged DNA and recruits other proteins or enzymes.
	
	Replication	Protein involved in the DNA replication process.
	
	Telomeric Protein	Protein that binds telomere parts of a chromosome contributing to its stability.
	
	Zalpha	Protein that binds left-handed DNA.

Transcription Factor	Alpha Helix	Protein that interacts with DNA mainly through α-helices.
	
	Alpha/Beta	Protein interacting with DNA through α-helices and β-strands.
	
	Beta Sheet	Protein that interacts with DNA mainly through β-sheets.
	
	Helix Turn Helix	Protein that contains the HtH motif according to the information available in PDB. It includes those proteins containing the "winged helix" domain.
	
	Ribbon/Helix/Helix	Protein that contains the RHH fold according to the information available in PDB.
	
	Zinc Coordinating	Protein that coordinates the metal in order to bind DNA.
	
	Zipper Type	Protein that contains the zipper motif, including the helix-loop-helix one.

**Figure 2:**
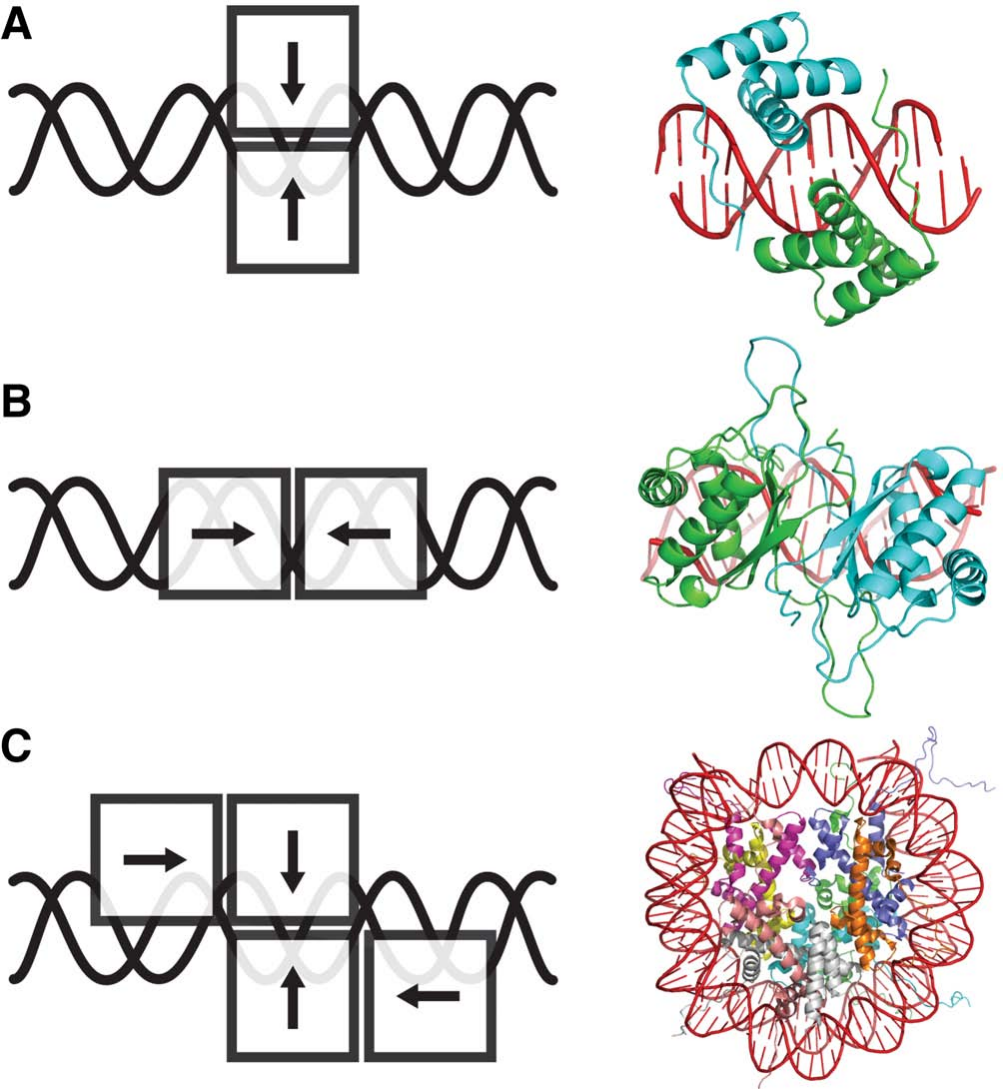
**Protein-protein interaction modes with DNA**. Three modes of protein-protein interaction with DNA are defined, according to the direction and the axis of the DNA helix. (A) Mode 1, the direction of the protein interaction and the double helix axis are orthogonal. (B) Mode 2, the direction of the interaction is parallel to the double helix axis. (C) Mode 3, both previous modes are observed at the same time. In the Mode 3 example, the histone core shown is the only instance of this case in the current version of the database. The histone core presents a set of proteins interacting with each other, thus making up a continuous interface with DNA. Additionally, a Mode 0 to assign those interfaces with only one protein has also been defined (not shown).

In addition to this classification, the following protein features were also recorded for each entry in the database: the number of protein monomers (or chains) interacting with DNA and being part of the interface; the type of multimerization, that accounts for whether the proteins are homomultimeric, heteromultimeric, or if both types can be found simultaneously at the interface; and the type of protein-protein interactions in the interface, which represents the way multimeric proteins interact with each other when contacting the DNA. In this regard, we have defined three interaction modes: Mode 1, where the direction of the protein interaction and the double helix axis are orthogonal (Figure 2, panel A); Mode 2, where the direction of the interaction is parallel to the double helix axis (Figure 2, panel B); and Mode 3, where both previous modes of interaction are observed at the same time (Figure 2, panel C).

### DNA features

**Figure 3:**
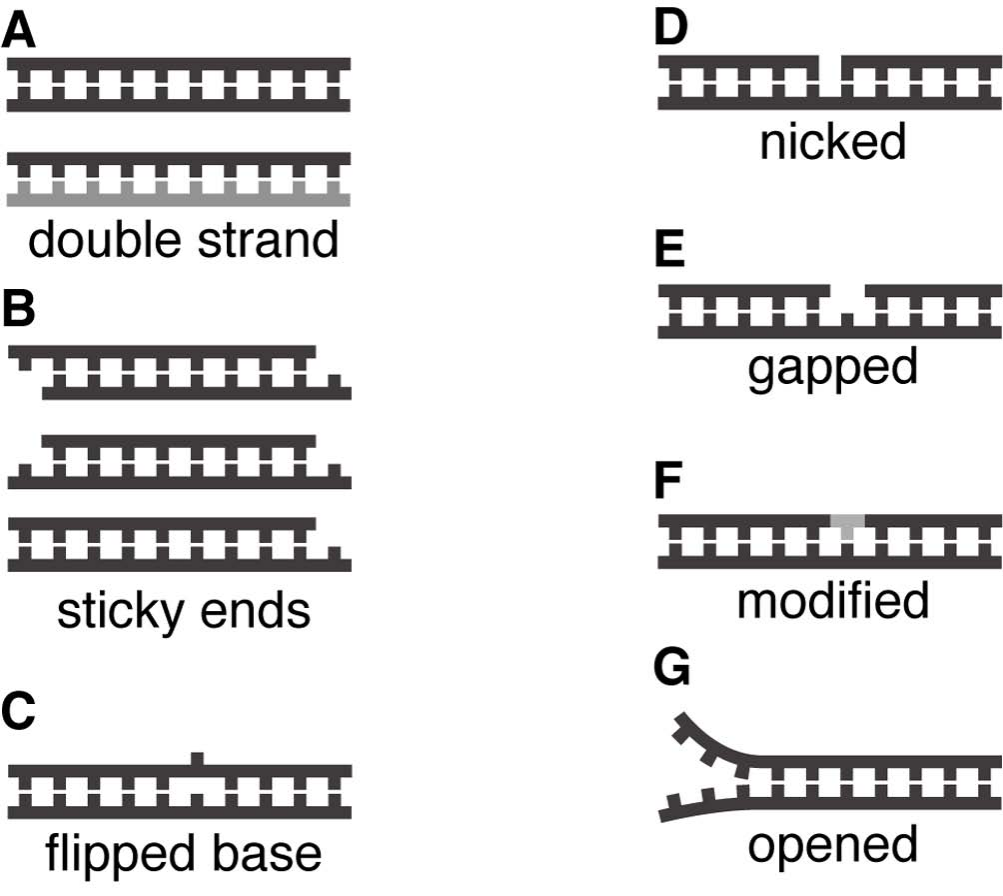
**DNA features**. Several DNA features has been defined. (A) Double strand or single strand in the asymmetric unit. This is useful to identify those interfaces coming from the reconstruction of the biological unit. (B) Sticky ends were defined based on the specific strands and the number of free bases at their ends. (C) Presence of flipped bases. (C) Existance of nicked DNA. (E) Existance of gapped DNA. (F) Presence of modified or non-standard DNA bases. (G) Presence of opened or unpaired bases at the DNA ends. Although not depicted here, left-handed DNA conformation was also recorded (Z-DNA).

In addition to the PDB and protein features described above, several DNA features were also recorded for each entry (Figure 3). These features include: double/single strand (Figure 3, panel A), where in the current version of the database the only possible types are double strand or single strand in the asymmetric unit (because the database contains only double strand DNA); sticky ends (Figure 3, panel B), that represent the unpaired bases at the end of the double-stranded DNA; flipped base (Figure 3, panel C), which represents whether the DNA has flipped bases; nicked DNA (Figure 3, panel D), that accounts for whether the DNA molecule has a broken phosphodiester bond in one or both strands; gapped DNA (Figure 3, panel E), which denotes if the DNA lacks one or more bases in the middle of one strand; modified DNA (Figure 3, panel F), that indicates if the DNA molecule contains chemically-modified or non-standard bases; open DNA (Figure 3, panel G), that occurs when a DNA molecule has unpaired canonical Watson-Crick bases toward the ends of the molecule; and Z-DNA, which represents whether the DNA molecule is in left-handed conformation or not.

It is worth noting that these features are applicable to the DNA structure present in the PDB file, so that two different interfaces coming from the same complex will normally share the same DNA features, unless the complex has more than one DNA molecule, which for example is the particular case of the structure with PDB code 1iaw.

### Interface features and effective interactions

These features take into account detailed atomic characteristics involving the interaction between protein and DNA. All these features rely on the results obtained after applying a recently described methodology [13] that allows the extraction of the effective atomic interactions between two molecules forming a complex. Briefly, a given atom in a DNA or protein structure can have many neighbour atoms in three-dimensional space, which are typically defined by setting up a fixed maximum distance threshold. In the absence of additional definitions, all these atoms found in such neighbourhood (*ie*. within the sphere defined by its centre and its radius) are considered to be interacting with it. However, by using this simple approach, many indirect interactions that in fact are shielded by other atoms and thus could not be relevant from a physical point of view, will still be included in the analysis. In order to avoid this problem, additional restraints are introduced so as to select only the direct interactions between two atoms. Direct or effective interactions are defined as those atom-atom interactions that are not shielded or masked by any other atom in three-dimensional space. A simple geometric algorithm was developed to assess the shielding effect that any atom has on the interaction of two other atoms [13].

**Figure 4:**
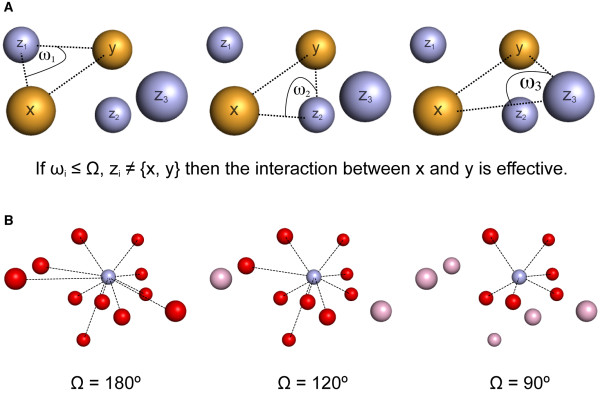
**Definition of effective atomic interactions**. (A) To determine if the interaction between DNA atom X and protein atom Y is effective, all other atoms inside the X interacting sphere (Z_i _atoms) are evaluated by comparing each ω_i _angle (*i.e*. the angle between atoms X, Z_i _and Y) with a defined shielding angle value Ω. If all the ω_i _angles observed are smaller than Ω, then the interaction between X and Y is defined as effective. (B) Three-dimensional view of three example interacting spheres of X, which only differ in the value of Ω. Red balls represent those protein atoms interacting effectively with DNA atom X. Pink balls represent protein atoms not interacting effectively with X, since they are shielded by other atoms inside the interacting sphere of X, according to the Ω value defined and used. A definition of Ω = 90° commonly captures the first interacting atom shell, while using Ω = 180° all the interactions observed inside the contacting sphere are considered as effective. To build this database a value of 90° for Ω was adopted.

Based on this methodology, it is possible to classify each interaction observed at a given protein-DNA interface as being either effective or not (Figure 4). Here we have used this methodology to select for each interface those protein-DNA atom pairs that interact effectively at a maximum distance of 7 Å. The atoms selected this way make up a cloud of points in three-dimensional space that we define as the effective interface, where every protein atom has an effective interacting DNA counterpart.

**Figure 5:**
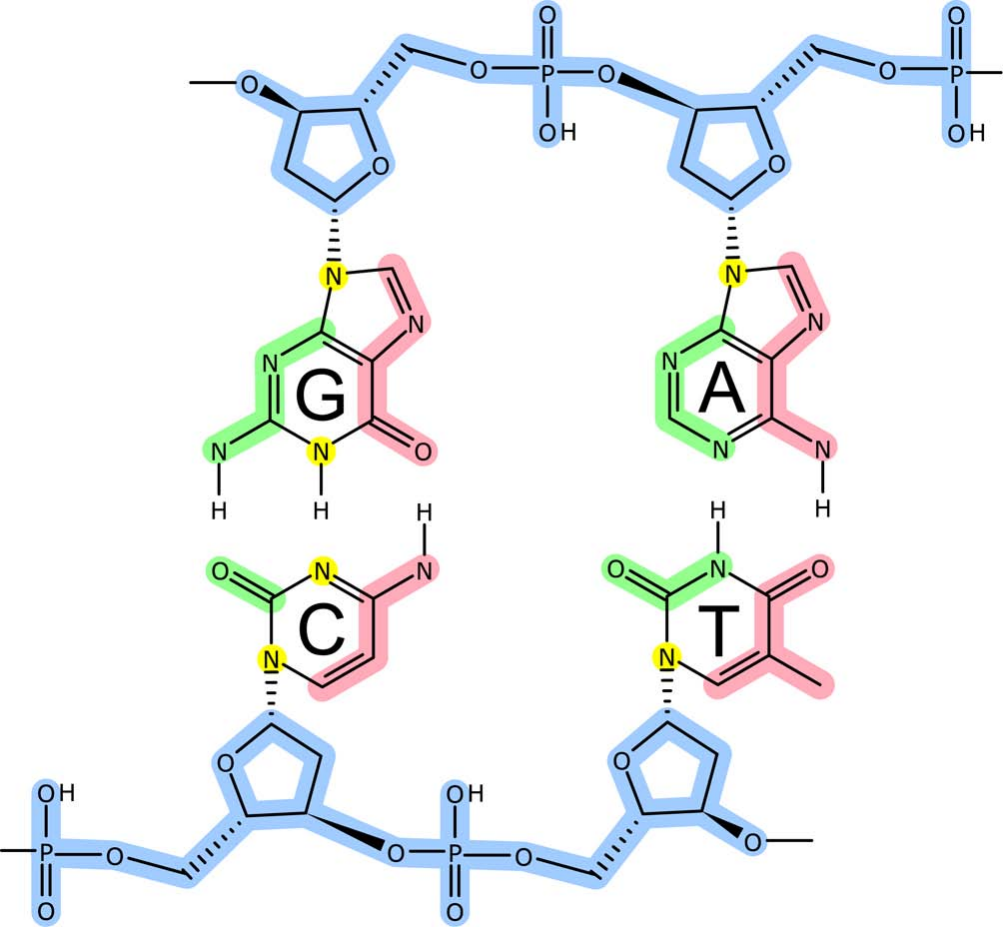
**Classification of DNA atoms**. All the interactions occurring in an effective interface were classified according to the chemical/structural/groove position of the DNA atoms. Pink-highlighted atoms were classified as being part of the major groove, green-highlighted atoms belong to the minor groove, blue-highlighted atoms belong to the backbone and sugar, and yellow-highlighted atoms were classified as not assigned since they are in an ambiguous location.

As each atomic pairwise interaction in the effective interface is identified in detail, we have classified them according to their position in the DNA grooves and to their physical-chemical nature. The specific groove location of DNA atoms was assigned according to the classical definition in B-DNA [14,15]. Thus, there are DNA atoms in the major groove, minor groove, backbone (phosphate and sugar), and atoms assigned to any location (*i.e*. ambiguous location) (Figure 5). Regarding the physical-chemical nature of the interacting atom pair we have defined the following five major classes of interactions (Table 2): **CHb**, interactions that resemble canonical H-bond (*i.e*. where the heavy atoms are either nitrogen or oxygen) [16]; **SHb**, interactions that resemble H-bonds with Sulphur [17]; **CHO**, contacts that resemble H-bonds of type CH··· O [18]; **Ion**, are interactions between charged atoms (*i.e*. any interaction where the protein atom is either NZ from lysine, NH1, NH2 from arginine, OD1, OD2 from aspartic acid, OE1, OE2 from glutamic acid, and the DNA atom is any oxygen from the phosphate groups); and **Hph**, contacts consisting of hydrophobic interactions. These five interaction classes in turn have subcategories that take into account the atom identity, the position of the atoms in both residues (*i.e*. edge, sidechain or backbone) and whether the atoms are donor or acceptor in the case they constitute an H-bond (Table 2). The total number of interaction types defined by using this procedure is 19, with an additional subcategory that is used to classify those interactions that do not belong to any of the types defined (*i.e*. not assigned).

**Table 2:** Definition of interaction classes and types

Class	Type	Detail
CHb	1	DBE-PSC: N_A _- N_D_
	
	2	DBE-PSC: N_A _- O_D_
	
	3	DBE-PSC: O_A _- N_D_
	
	4	DBE-PSC: O_A _- O_D_
	
	5	DBE-PSC: N_D _- O_A_
	
	6	DBE-PBB: N_A _- N_D_
	
	7	DBE-PBB: N_D _- O_A_
	
	8	DBE-PBB: O_A _- N_D_
	
	9	DBB-PSC: O_A _- N_D_
	
	10	DBB-PSC: O_A _- O_D_
	
	11	DBB-PBB: O_A _- N_D_

SHb	12	DBB-PSC: O_A _- S_D_
	
	13	DBE-PSC: N_A _- S_D_
	
	14	DBE-PSC: O_A _- S_D_
	
	15	DBE-PSC: N_D _- S_A_

CHO	16	DBE-PSC: C_D _- O_A_
	
	17	DBE-PSB: C_D _- O_A_

Ion	18	Ionic bond: (+)··· (-)

Hph	19	C - C
	
	20	Not assigned

The nomenclature of the abbreviations used in this Table is the following: DBE, DNA Base edge; DBB, DNA Backbone; PSC, Protein Sidechain; PBB, Protein Backbone; X_A_, Acceptor; X_D_, Donor; O, Oxygen; N, Nitrogen; S, Sulphur; C, Carbon. See text for more information.

### Database redundancy, clustering and representative members

To remove obvious redundancy from this database, we have performed two independent clustering/grouping schemes: 1) a clustering based on protein sequence identity and fraction of aligned regions for those proteins that form part of the interface with DNA; and 2) a clustering based on the effective interactions observed for each interface between proteins and DNA.

The sequence-based clustering was obtained by aligning all the protein sequence chains that interact with DNA in a pairwise fashion. Sequences were clustered in groups according to a length coverage threshold of 90% and percentage sequence identity of 70%, using blastclust software. Therefore, two protein-DNA interfaces were clustered together if any two protein chains found at the two interfaces shared more than 70% sequence identity for at least 90% of the length of both sequences. A total of 246 non-redundant interfaces, out of the initial 922 entries, were obtained with this procedure.

The interface-based clustering was obtained by calculating a dissimilarity measure between all different interface pairs. This measure is described in detail in the database web site ("About" section). This dissimilarity measure ranges between 0, for identical interfaces, and 1, for two interfaces that have no interactions in common. Using this measure, a difference table was built for all possible interface pairs and hierarchical clustering carried out with the group average algorithm. We used a threshold of 0.25 to define the non-redundant groups. This means that two interfaces were clustered together if they had more than 75% of their effective interactions in common. A total of 671 non-redundant interfaces, out of the initial 922 entries, were obtained with this procedure.

The detailed list of representative interfaces, as well as the members belonging to each group, are available online at the database web site for both clustering/grouping schemes. Additionally, complex queries for each clustered set can be launched through the advanced search option of the web interface. Finally, a set of PDB files of the representative members for each clustered set, the detailed interface data, as well as other related sets can be downloaded directly from the database web site ("Download" section).

## Utility and Discussion

### Web User Interface

PDIdb was built using the PHP framework Symfony, the AJAX technology and the MySQL database management system. The database can be accessed through Internet at http://melolab.org/pdidb and several options are available (described below).

**Figure 6:**
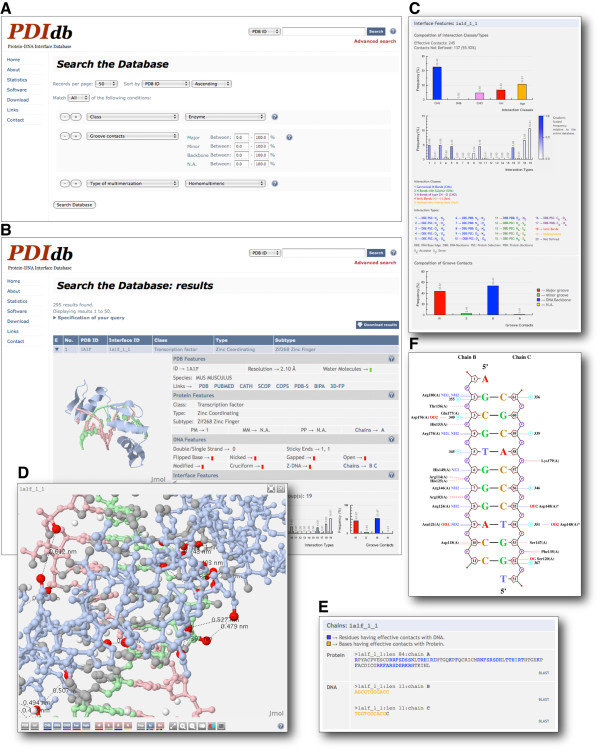
**Web user interface of PDIdb**. (A) The database search engine. There are two search modes: basic and advanced. A basic search can be carried out by entering a PDB code or a keyword. The advanced search allows the user to make more complex queries through a dynamic expanding search form, by combining many subqueries that search all fields available in the database. (B) The result of the query is a table where each row shows basic information about the interface. When the user clicks on it, the row expands and a new table with detailed information, 2D and 3D molecular graphics is displayed. (C) For each interface, this information includes the protein and DNA features, as well as the detailed composition of the atomic contacts. (D) The user can inspect graphical information at both the sequence and structure level. A Jmol applet is available to explore the structure and the atomic interactions conforming the interface in three-dimensional space. (E) Sequences highlighting the contacting residues are available in FASTA format for further analysis (*e.g*. BLAST). (F) The protein-DNA complex can be also explored by means of NUCPLOT graphs, which map onto a plane direct or water-mediated hydrogen bonds between aminoacids and nucleotides.

The core of the database is its search engine. There is two search modes: basic and advanced. A basic search can be carried out by entering a PDB code or a keyword. Meanwhile, the advanced search allows the user to make more complex queries through a dynamic expanding search form, by combining many subqueries to search all field value options available in the database (Figure 6, panel A). We must point out that the options and search fields are built on the fly and immediately provided to the user by quickly interrogating the information currently available in the database.

The result of the query is a table where each row shows basic information about the interface. This basic information consists of: the PDB code of the original structure, the unique PDIdb ID and the protein classification. When the user clicks on a given entry, the row expands and a new table with detailed information about the corresponding protein-DNA interface is displayed (Figure 6, panel B). For each interface, the detailed information is divided into different sections, which are described next. The *PDB Features *section displays information concerning the original structure such as the resolution, source species and whether the structure has water molecules (Figure 6, panel B). This section also contains direct links to the following important and related databases: PDB, PUBMED, CATH, SCOP, COPS, PDBSum [19], BIPA and 3D-Footprint. The *Protein Features *section shows the classification of the protein part of the interface and all features previously described above (see previous section). Analogously, the *DNA Features *section displays graphically all DNA features previously described above (see previous section). Both *Protein and DNA Features *sections have a link through the chain ID (Figure 6, panel B), which displays a pop-up window with the sequences in FASTA format, highlighting the contacting residues and linking the sequences to a query ready for BLAST analysis at the NCBI website server (Figure 6, panel E). The *Interface Features *section describes the detailed composition of the atomic contacts in the effective interface (Figure 6, panel B). This information is displayed as bar plots and includes the groove contacts as well as the interaction classes and types (see previous section above). When a plot is clicked, a pop-up window opens showing the detailed view of the graphs and the respective legends (Figure 6, panel C). The *Interface Features *section also includes the display of a NUCPLOT graph [20], which maps onto a plane direct or water-mediated hydrogen bonds between aminoacids and nucleotides as determined by HBPLUS [21] (Figure 6, panel F), and a link to a pop-up displaying all interfaces where protein sequences belong to the same sequence group or cluster (see above). Independent files containing the parsed PDB structure, the 3D coordinates of all atoms being part of the effective interface in PDB format and the detailed information of the interaction types found at the effective interface in plain text format, can be downloaded in the *Download *section.

Next to the sections described above, a Jmol [22] applet is available to explore the structure and the atomic interactions constituting the effective interface in three-dimensional space. Several molscripts were created in order to survey the groove contacts as well as the interaction classes and types through this applet. We have also included coordinate files containing the middle points for each pair of contacting atoms for further comparison purposes. For a detailed view of the structure, a pop-up window of the applet can also be launched by the user. This pop-up window includes more options such as drawing lines and showing distances between the contacting atoms, zooming, stereo and anaglyph views (Figure 6, panel D). Console driven, user custom Jmol scripts can also be directly launched from here.

### Database Statistics

In the current version of the database there are a total of 922 entries, which will be doubtless increased with future updates. By considering the classification of the protein part of the interfaces introduced in this report, out of the total entries, 528 (57%) are categorized as *Enzyme*, whose main types are Polymerase and Endonuclease (37% and 29%, respectively); 295 (32%) belong to the class *Transcription Factor*, which contains the main types Helix-turn-Helix (42%) and Zinc Coordinating (20%); and 99 (11%) are classified as *Structural/DNA Binding Protein*, whose most abundant category is the type DNA Packaging (34%), which in turn includes the subtypes Histone and Histone-like proteins. A graphical representation of these figures can be also found at the web site under the menu Statistics.

The multimerization state in the interfaces shows that 608 (66%) proteins are monomeric, 294 (32%) are dimeric, and the rest with 3 or more monomeric units. Out of the interfaces with multimeric proteins, 261 (83%) are homomultimeric and 41 (13%) are heteromultimeric.

As to the DNA features, 427 (46%) interfaces out of the total have DNA molecules with no sticky ends, 266 (29%) have sticky ends in both strands, 200 (22%) have sticky ends in one strand at one end, and the remainder (3%), have sticky ends at both ends in one strand; 150 (16%) out of the total number of interfaces have flipped bases; 39 (4%) structures have nicked DNA, 22 of which at both strands; 49 (5%) interfaces have gapped DNA, while 62 (7%) have open DNA; 278 (30%) have at least a modified or non-standard base; and finally, only 14 (2%) interfaces have left-handed DNA.

When effective contacts are regarded, on average all interfaces have 296.3 total contacts, 18.11% of which occurs with the major groove, 7.92% with the minor groove, and 72.92% with the backbone (phosphate and sugar). Concerning interaction classes, on average 15.83% out of all interactions can be classified as canonical hydrogen bonds, 1.24% are hydrogen bonds of the class CH··· O, 5.56% are classified as ionic bonds, and 16.49% are considered as hydrophobic interactions. Out of all interactions, 60.79% on average cannot be assigned to one of the 19 defined classes. The detailed description of these statistics can be found in Additional file 2.

### Download Data and Software

At the web site, the complete data that constitute the database in raw format is also available for download. The data includes the corresponding parsed PDB files, the effective atomic interfaces (in PDB format) and the relevant protein and DNA features, along with other useful detailed data calculated from the effective interfaces.

The database is also accompanied with computer software. This specialized software was developed to extract the effective atomic interfaces of protein-DNA complexes and to classify their interaction types. As previously mentioned, the analysis of effective atomic interactions is useful to better characterize and describe the common and unique features present at different complex interfaces, which in turn could help to elucidate the key specificity determinants involved in a particular protein-DNA recognition process.

The software is composed of main and supporting applications written in C++ for command-line execution. These applications are highly customizable, use PDB files as input and include different options. There are two main applications: one is used to analyse any kind of atom-atom contact, where the user can define atom types, centroids (*i.e*. average 3D coordinates of several atom positions) and distance ranges; in the other application, the user can also define the type of interaction that any pair of atoms may have (*e.g*. hydrogen bonds, hydrophobic interactions, etc.). These analyses could be done including water molecules at the time of detecting the effective atomic interactions at the complex interface. According to the options selected, the output of the software can be the contact matrices, files with detailed information about interactions and/or molscripts to visualize the interactions. The components and modules of this software are fully documented in the software distribution.

### Future Directions

Several improvements are going to be introduced in the near future to PDIdb. We intend to include more interaction types such as cation-π contacts [23], interactions with cation/anion ligands, a more detailed description of water-mediated interactions and the inclusion of non-standard or modified bases in the interfaces. We also plan to include more DNA features such as the groove lengths and the electrostatic potential at the interface [24]. At the moment, we are doing a deeper analysis of the data kept in the database, including a structural clustering of the effective interfaces, which we hope results in a new classification of protein-DNA complexes. Finally, we are also developing a new interface graphical representation that will include the effective interaction classes.

## Conclusions

The existing resources and databases advocated to the study of protein-DNA interactions, as well as the increasing amount of protein-DNA complexes available at the PDB, are a result of the great interest for better understanding the molecular recognition process between DNA and proteins. The PDIdb represents a novel repository that disaggregates three-dimensional protein-DNA structure complexes toward the level of interface, which constitutes a simultaneous view of both the protein and DNA parts of the interacting complex. The most important added value of this database is that most of the information recorded has been manually curated. This means that each original PDB file has been visually inspected, dissected in interfaces, analysed in its constituent parts and finally classified by using simple but well-defined criteria. The automated process involved in the building of this database has only to do with the obtention of the effective interface and the classification of the interactions. When we relate and integrate both kind of information, a great diversity of binding modes are seen at a glance, which certainly needs a deeper future analysis by properly mining the information available here.

Consequently, this database will be useful to those people working in the fields of prediction of transcription factor binding sites in DNA, study of specificity determinants that mediates different enzyme recognition events, engineering and design of new transcription factors with distinct binding specificity and affinity and many other applications. It is important to mention that due to its friendly and easy-to-use web user interface, this database might also serve educational and teaching purposes.

## Availability and Requirements

The database and software are freely accesible to academic and non-academic users from our web site located at: http://melolab.org/pdidb.

The complete set of experimental data used in this work and the results obtained are available at our web site: http://protein.bio.puc.cl/sup-mat.html

## Authors' contributions

FM conceived the project. TN wrote all required computer software to build the database and processed each entry in a semiautomated fashion. TN also designed and implemented the web interface and manually inspected/curated the complete database content. Both authors wrote this manuscript.

## Supplementary Material

Additional file 1 **Protein-DNA interactions resources and databases**. A list of currently available resources with data about protein-DNA complexes and their corresponding references. Click here for file

Additional file 2 **Detailed statistics of effective atomic interfaces**. Figures and percentages of effective interactions obtained from protein-DNA complexes, categorized according to groove contacts and interactions classes. Click here for file
