# Supplementary material for: The Protein-DNA Interface database
Source: BMC Bioinformatics. 2010 May 18;11:262. doi: 10.1186/1471-2105-11-262 (PMC2885377; doi:10.1186/1471-2105-11-262)
Supplement: Additional file 1 — Protein-DNA interactions resources and databases. A list of currently available resources with data about protein-DNA complexes and their corresponding references. [file 1471-2105-11-262-S1.DOC]

**Additional file 1**. Protein-DNA interactions resources and databases*

| **Name** | **Type** | **Main Feature** | **Published** | **Last Update**** | **Address** | **Ref.** |
| --- | --- | --- | --- | --- | --- | --- |
| 3d-footprint | DB | Collection of PWM extracted from structure. | 2010 | Feb 2010 | http://floresta.eead.csic.es/3dfootprint | [1] |
| 3DNA | S | Tool for extracting DNA structure parameters. | 2003 | - | http://rutchem.rutgers.edu/~xiangjun/3DNA | [2] |
| AANT | DB | Collection of amino acid-nucleotide interactions derived from experimentally determined protein–nucleic acid structures. | 2004 | ? | http://aant.icmb.utexas.edu/ | [3] |
| BindN | S | SVM-based tool for prediction of DNA and RNA binding residues in protein. | 2006 | - | http://bioinfo.ggc.org/bindn/ | [4] |
| BIPA | DB | Database for protein–nucleic acid interactions in 3D structures. It provides various features of protein-nucleic acid interfaces. | 2009 | Aug 2009 | http://mordred.bioc.cam.ac.uk/bipa | [5] |
| Curves+ | S | Tool for extracting DNA structure parameters. | 2009 | - | http://gbio-pbil.ibcp.fr/Curves_plus | [6] |
| DNABindR | S | Web server for identification of DNA binding residues in protein sequences. | 2006 | - | http://turing.cs.iastate.edu/PredDNA/ | [7] |
| DP-Bind | S | Web-server that takes a sequence of a DNA-binding protein and predicts residue positions involved in interactions with DNA. | 2007 | - | http://lcg.rit.albany.edu/dp-bind/ | [8] |
| hPDI | DB | Database that holds experimental protein–DNA interaction data for humans identified by protein microarray assays. | 2010 | ? | http://bioinfo.wilmer.jhu.edu/PDI/ | [9] |
| IAlign | S | Software to align protein-DNA interfaces based on a matrix score. | 2005 | - | http://luna.bioc.columbia.edu/honiglab/software/IAlign/ | [10] |
| JASPAR | DB | Open-access database for eukaryotic transcription factor binding profiles. | 2004 | 2010 | http://jaspar.genereg.net | [11] |
| NDB | DB | The nucleic acid database. | 1992 | Feb 2010 | http://ndbserver.rutgers.edu/ | [12] |
| NPIDB | DB | Database containing information derived from structures of DNA-protein and RNA-protein complexes extracted from PDB. | 2007 | Jul 2008 | http://mouse.belozersky.msu.ru/NPIDB/ | [13] |
| NUCPLOT | S | Programme to generate schematic diagrams  of protein–nucleic acid interactions. | 1997 | - | http://www.biochem.ucl.ac.uk/bsm/nucplot.html | [14] |
| PDA | S | Automatic programme for the analysis of protein-DNA complex structures. | 2009 | - | http://bioinfozen.uncc.edu/webpda/ | [15] |
| PDBSum | DB | Web-based database of summaries and analyses of all PDB structures. | 1996 | Feb 2010 | http://www.ebi.ac.uk/pdbsum/ | [16] |
| ProNIT | DB | Database that collects experimentally observed binding data from the literature. It contains several important thermodynamic data for protein-nucleic acid binding. | 2001 | Jan 2010 | http://gibk26.bse.kyutech.ac.jp/jouhou/pronit/pronit.html | [17] |
| ProNuC | DB | Database containing structural data of protein-nucleic acid complex. The data are classified according to recognition motif of proteins and DNA forms involved in the complex. | 1998 | Jul 2006 | http://gibk26.bse.kyutech.ac.jp/jouhou/pronuc/pronuc.html | [18] |
| ProtNA-ASA | DB | Database that combines the data on conformational parameters of nucleic acids and accessible surface area of nucleic acid atoms in protein-DNA/RNA complexes. | 2009 | ? | http://www.protna.bio-page.org/ | [19] |
| Tfmodeller | S | Programme for comparative modelling of protein-DNA complexes. | 2007 | - | http://maya.ccg.unam.mx/~tfmodell/ | [20] |
| TRANSFAC | DB | Private database for eukaryotic transcription factor binding profiles. | 1996 | 2009 | http://www.biobase-international.com/pages/index.php?id=transfac | [21] |
| ZifBase | DB | Collection of various natural and engineered zinc finger proteins, containing sequence features and linked to their structures. | 2009 | ? | http://web.iitd.ac.in/~sundar/zifbase/ | [22] |

* This table and the listed links can be also accessed at the web site of the database <http://melolab.org/pdidb>

** Until the date this report was published.

**REFERENCES**

1. Contreras-Moreira B: **3D-footprint: a database for the structural analysis of protein-DNA complexes.** *Nucleic Acids Res* 2010, **38**:D91-D97.

2. Lu XJ, Olson WK: **3DNA: a software package for the analysis, rebuilding and visualization of three-dimensional nucleic acid structures.** *Nucleic Acids Res* 2003, **31**:5108-5121.

3. Hoffman MM, Khrapov MA, Cox JC, Yao J, Tong L, Ellington AD: **AANT: the Amino Acid-Nucleotide Interaction Database.** *Nucleic Acids Res* 2004, **32**:D174-D181.

4. Wang L, Brown SJ: **BindN: a web-based tool for efficient prediction of DNA and RNA binding sites in amino acid sequences.** *Nucleic Acids Res* 2006, **34**:W243-W248.

5. Lee S, Blundell TL: **BIPA: a database for protein-nucleic acid interaction in 3D structures.** *Bioinformatics* 2009, **25**:1559-1560.

6. Lavery R, Moakher M, Maddocks JH, Petkeviciute D, Zakrzewska K: **Conformational analysis of nucleic acids revisited: Curves+.** *Nucleic Acids Res* 2009, **37**:5917-5929.

7. Yan C, Terribilini M, Wu F, Jernigan RL, Dobbs D, Honavar V: **Predicting DNA-binding sites of proteins from amino acid sequence.** *BMC Bioinformatics* 2006, **7**:262.

8. Hwang S, Gou Z, Kuznetsov IB: **DP-Bind: a web server for sequence-based prediction of DNA-binding residues in DNA-binding proteins.** *Bioinformatics* 2007, **23**:634-636.

9. Xie Z, Hu S, Blackshaw S, Zhu H, Qian J: **hPDI: a database of experimental human protein-DNA interactions.** *Bioinformatics* 2010, **26**:287-289.

10. Siggers TW, Silkov A, Honig B: **Structural alignment of protein--DNA interfaces: insights into the determinants of binding specificity.** *J Mol Biol* 2005, **345**:1027-1045.

11. Sandelin A, Alkema W, Engstrom P, Wasserman WW, Lenhard B: **JASPAR: an open-access database for eukaryotic transcription factor binding profiles.** *Nucleic Acids Res* 2004, **32**:D91-D94.

12. Berman HM, Olson WK, Beveridge DL, Westbrook J, Gelbin A, Demeny T, Hsieh SH, Srinivasan AR, Schneider B: **The nucleic acid database. A comprehensive relational database of three-dimensional structures of nucleic acids.** *Biophysical journal* 1992, **63**:751-759.

13. Spirin S, Titov M, Karyagina A, Alexeevski A: **NPIDB: a database of nucleic acids-protein interactions.** *Bioinformatics* 2007, **23**:3247-3248.

14. Luscombe NM, Laskowski RA, Thornton JM: **NUCPLOT: a program to generate schematic diagrams of protein-nucleic acid interactions.** *Nucleic Acids Res* 1997, **25**:4940-4945.

15. Kim R, Guo JT: **PDA: an automatic and comprehensive analysis program for protein-DNA complex structures.** *BMC Genomics* 2009, **10 Suppl 1**:S13.

16. Laskowski RA, Hutchinson EG, Michie AD, Wallace AC, Jones ML, Thornton JM: **PDBsum: a Web-based database of summaries and analyses of all PDB structures.** *Trends Biochem Sci* 1997, **22**:488-490.

17. Prabakaran P, An J, Gromiha MM, Selvaraj S, Uedaira H, Kono H, Sarai A: **Thermodynamic database for protein-nucleic acid interactions (ProNIT).** *Bioinformatics* 2001, **17**:1027-1034.

18. An J, Nakama T, Kubota Y, Sarai A: **3DinSight: an integrated relational database and search tool for the structure, function and properties of biomolecules.** *Bioinformatics* 1998, **14**:188-195.

19. Tkachenko MY, Boryskina OP, Shestopalova AV, Tolstorukov MY: **ProtNA-ASA: Protein-nucleic acid structural database with information on accessible surface area.** *International Journal of Quantum Chemistry* 2009, **110**:231-232.

20. Contreras-Moreira B, Branger PA, Collado-Vides J: **TFmodeller: comparative modelling of protein-DNA complexes.** *Bioinformatics* 2007, **23**:1694-1696.

21. Wingender E, Dietze P, Karas H, Knuppel R: **TRANSFAC: a database on transcription factors and their DNA binding sites.** *Nucleic Acids Res* 1996, **24**:238-241.

22. Jayakanthan M, Muthukumaran J, Chandrasekar S, Chawla K, Punetha A, Sundar D: **ZifBASE: a database of zinc finger proteins and associated resources.** *BMC Genomics* 2009, **10**:421.
