# Supplementary material for: The Protein-DNA Interface database
Source: BMC Bioinformatics. 2010 May 18;11:262. doi: 10.1186/1471-2105-11-262 (PMC2885377; doi:10.1186/1471-2105-11-262)
Supplement: Additional file 2 — Detailed statistics of effective atomic interfaces. Figures and percentages of effective interactions obtained from protein-DNA complexes, categorized according to groove contacts and interactions classes. [file 1471-2105-11-262-S2.DOC]

**Additional file 2**. Detailed statistics of effective atomic interfaces.

| **Class** | **Type** | **Freq.** | **Contacts** | **Groove contacts** | | | | **Interaction classes** | | | | | |
| --- | --- | --- | --- | --- | --- | --- | --- | --- | --- | --- | --- | --- | --- |
|  |  |  |  | **W** | **S** | **B** | **N** | **CHb** | **SHb** | **CHO** | **Ion** | **Hph** | **NA** |
| Enzyme | Dioxygenase | 6 | 197.5 (75.3) | 18.50 (3.06) | 9.14 (7.70) | 69.70 (9.71) | 2.66 (1.84) | 12.01 (3.51) | 0.77 (0.85) | 0.33 (0.51) | 7.23 (1.63) | 21.34 (3.15) | 58.31 (1.50) |
|  | Endonuclease | 151 | 432.2 (155.0) | 24.16 (8.40) | 5.39 (4.10) | 69.70 (6.39) | 0.74 (1.37) | 16.84 (3.15) | 0.04 (0.14) | 1.67 (0.80) | 4.99 (1.84) | 15.69 (3.38) | 60.78 (3.05) |
|  | Excisionase | 3 | 142.3 (82.2) | 18.71 (2.24) | 3.71 (3.22) | 77.58 (2.55) | 0.00 (0.00) | 15.52 (0.81) | 0.00 (0.00) | 2.43 (1.44) | 12.89 (6.72) | 8.74 (4.87) | 60.43 (4.89) |
|  | Glucosyltransferase | 14 | 165.2 (35.8) | 24.58 (8.73) | 13.83 (4.02) | 57.89 (9.07) | 3.70 (1.04) | 14.77 (3.63) | 0.04 (0.15) | 1.34 (0.87) | 5.46 (2.35) | 16.95 (4.16) | 61.44 (2.88) |
|  | Glycosylase | 86 | 168.1 (54.3) | 9.46 (4.92) | 14.22 (4.70) | 73.61 (9.18) | 2.70 (1.50) | 16.31 (4.56) | 0.18 (0.46) | 0.49 (0.68) | 3.04 (1.85) | 17.00 (3.87) | 62.98 (3.89) |
|  | Helicase | 2 | 298.0 (12.7) | 16.14 (1.64) | 14.92 (0.55) | 65.07 (1.49) | 3.87 (0.40) | 15.91 (1.46) | 0.00 (0.00) | 1.16 (0.66) | 2.49 (1.08) | 23.03 (2.17) | 57.40 (1.03) |
|  | Ligase | 1 | 582.0 | 2.75 | 6.70 | 90.38 | 0.17 | 17.35 | 0.00 | 0.17 | 6.70 | 14.60 | 61.17 |
|  | Methyltransferase | 32 | 287.9 (74.9) | 24.82 (5.22) | 8.59 (2.33) | 64.80 (5.57) | 1.79 (1.60) | 15.62 (2.66) | 0.18 (0.26) | 1.75 (0.77) | 5.57 (1.71) | 17.73 (4.10) | 59.15 (4.36) |
|  | Nuclease | 5 | 257.0 (100.2) | 15.09 (13.09) | 8.28 (1.94) | 76.58 (11.36) | 0.06 (0.14) | 17.52 (4.89) | 0.06 (0.14) | 1.82 (1.66) | 5.44 (2.15) | 19.09 (1.45) | 56.06 (2.66) |
|  | Photolyase | 5 | 158.8 (55.3) | 8.90 (4.81) | 9.58 (3.84) | 80.44 (6.90) | 1.08 (1.39) | 14.28 (4.27) | 0.00 (0.00) | 0.59 (0.42) | 5.68 (0.95) | 20.01 (4.63) | 59.44 (1.83) |
|  | Polymerase | 195 | 294.0 (92.0) | 9.09 (3.92) | 11.13 (6.21) | 78.13 (8.69) | 1.65 (1.20) | 15.83 (1.96) | 0.03 (0.10) | 0.32 (0.43) | 4.79 (2.23) | 15.94 (3.41) | 63.09 (2.30) |
|  | Recombinase | 15 | 622.1 (342.3) | 20.15 (3.05) | 7.09 (4.11) | 72.69 (2.33) | 0.07 (0.16) | 16.73 (1.12) | 0.16 (0.12) | 1.72 (1.19) | 5.38 (2.04) | 16.53 (0.98) | 59.47 (2.76) |
|  | Topoisomerase | 6 | 332.3 (40.4) | 13.71 (8.27) | 2.74 (1.22) | 83.54 (8.01) | 0.00 (0.00) | 16.78 (2.26) | 0.00 (0.00) | 1.02 (0.44) | 8.00 (0.93) | 13.54 (2.58) | 60.66 (1.02) |
|  | Translocase | 2 | 324.5 (4.9) | 26.04 (0.18) | 0.31 (0.00) | 73.65 (0.18) | 0.00 (0.00) | 16.65 (0.69) | 0.00 (0.00) | 1.39 (0.20) | 6.16 (0.78) | 17.72 (0.38) | 58.09 (0.67) |
|  | Transposase | 5 | 278.0 (43.5) | 30.56 (6.56) | 8.84 (4.31) | 59.68 (3.32) | 0.91 (0.48) | 14.86 (2.19) | 0.10 (0.22) | 1.28 (0.73) | 5.77 (2.08) | 17.52 (2.87) | 60.48 (3.90) |
| Structural/DNA Binding | Centromeric Protein | 1 | 409.0 | 20.29 | 5.38 | 74.08 | 0.24 | 17.36 | 0.00 | 1.96 | 4.65 | 17.85 | 58.19 |
|  | DNA Bending | 6 | 468.7 (12.5) | 6.44 (0.86) | 17.76 (1.51) | 75.16 (1.58) | 0.64 (0.19) | 17.83 (0.70) | 0.00 (0.00) | 0.32 (0.11) | 6.72 (0.55) | 17.02 (1.37) | 58.12 (1.59) |
|  | DNA Packaging | 34 | 623.7 (616.8) | 2.02 (1.44) | 15.92 (8.58) | 80.60 (10.06) | 1.46 (1.50) | 13.37 (3.73) | 0.02 (0.11) | 0.04 (0.07) | 5.92 (2.93) | 20.87 (6.57) | 59.79 (2.21) |
|  | Maintenance\|Protection | 15 | 233.6 (69.9) | 21.16 (6.80) | 9.74 (3.28) | 67.09 (4.66) | 2.01 (1.02) | 12.92 (1.76) | 0.10 (0.26) | 0.73 (0.30) | 4.30 (0.92) | 17.97 (1.69) | 63.98 (1.88) |
|  | Repair Protein | 8 | 330.8 (15.3) | 5.57 (0.95) | 12.42 (4.32) | 81.17 (4.51) | 0.84 (0.37) | 13.93 (1.58) | 0.03 (0.10) | 0.61 (0.28) | 6.31 (2.60) | 17.92 (2.31) | 61.19 (1.71) |
|  | Replication | 13 | 250.4 (155.8) | 29.88 (8.77) | 2.50 (3.75) | 67.39 (6.11) | 0.23 (0.55) | 20.42 (5.00) | 0.03 (0.10) | 1.09 (0.88) | 4.00 (1.63) | 15.64 (3.96) | 58.82 (2.12) |
|  | Telomeric Protein | 8 | 208.2 (139.7) | 27.05 (4.91) | 2.24 (1.57) | 70.71 (3.72) | 0.00 (0.00) | 17.90 (2.32) | 0.00 (0.00) | 1.59 (0.60) | 4.74 (2.15) | 15.10 (2.96) | 60.68 (3.00) |
|  | Zalpha | 14 | 79.4 (10.0) | 9.15 (2.59) | 0.62 (0.79) | 90.23 (2.59) | 0.00 (0.00) | 11.42 (2.09) | 0.00 (0.00) | 0.00 (0.00) | 6.82 (1.57) | 18.11 (3.31) | 63.66 (2.90) |
| Transcription factor | Alpha Helix | 13 | 243.3 (89.7) | 20.02 (11.74) | 9.54 (10.35) | 69.51 (5.95) | 0.93 (1.50) | 15.09 (2.45) | 0.25 (0.52) | 1.43 (1.83) | 5.99 (3.36) | 18.76 (5.83) | 58.49 (3.70) |
|  | Alpha\|Beta | 10 | 244.0 (77.3) | 21.53 (9.28) | 4.49 (6.06) | 73.84 (6.74) | 0.14 (0.33) | 14.60 (3.51) | 0.32 (0.69) | 1.41 (1.10) | 6.30 (2.43) | 16.19 (3.85) | 61.18 (4.49) |
|  | Beta Sheet | 53 | 293.2 (76.2) | 10.74 (11.93) | 12.32 (6.80) | 76.45 (5.66) | 0.48 (0.35) | 11.95 (6.67) | 0.05 (0.25) | 1.14 (1.47) | 5.64 (1.67) | 22.20 (7.37) | 59.02 (3.78) |
|  | Helix Turn Helix | 122 | 209.9 (109.2) | 25.07 (8.84) | 2.81 (3.28) | 71.99 (7.93) | 0.13 (0.26) | 15.72 (3.96) | 0.16 (0.52) | 2.18 (1.71) | 6.63 (3.12) | 15.69 (4.35) | 59.61 (3.99) |
|  | Ribbon\|Helix\|Helix | 14 | 185.9 (25.7) | 27.42 (9.05) | 0.18 (0.26) | 72.40 (8.88) | 0.00 (0.00) | 21.49 (3.96) | 0.00 (0.00) | 2.28 (0.78) | 6.21 (2.05) | 13.13 (3.52) | 56.90 (2.91) |
|  | Zinc Coordinating | 60 | 250.6 (83.5) | 30.81 (8.87) | 2.30 (2.81) | 66.64 (8.03) | 0.24 (0.49) | 18.28 (3.39) | 0.24 (0.91) | 2.07 (1.29) | 8.11 (2.36) | 12.55 (3.05) | 58.76 (4.19) |
|  | Zipper Type | 23 | 229.9 (31.6) | 37.77 (5.39) | 0.00 (0.00) | 62.16 (5.42) | 0.07 (0.16) | 13.98 (3.10) | 0.00 (0.00) | 3.53 (1.02) | 10.16 (4.34) | 15.00 (3.14) | 57.33 (5.74) |
| All Database | All Interfaces | 922 | 296.3 (195.5) | 18.11 (11.49) | 7.92 (6.75) | 72.92 (9.32) | 1.06 (1.39) | 15.83 (3.91) | 0.09 (0.38) | 1.24 (1.29) | 5.56 (2.75) | 16.49 (4.58) | 60.79 (3.80) |

Column Contacts displays the average of total effective contacts per Type. Columns Groove contacts and Interaction classes display the average percentage for each category (in parenthesis the standard deviations). W, major groove; S, minor groove; B, backbone; N, not assigned (ambiguous); CHb, canonical H-bonds; SHb, H-bonds with Sulphur; CHO, H-bonds of type CH···O; Ion, ionic bonds; Hph, hydrophobic interactions; NA, not assigned.
